# The PennAITech Experience: Promoting Use of Artificial Intelligence to Support Aging

**DOI:** 10.1093/geroni/igaf122.1522

**Published:** 2025-12-31

**Authors:** George Demiris

**Affiliations:** University of Pennsylvania, Philadelphia, Pennsylvania, United States

## Abstract

The overarching goal of the Penn Artificial Intelligence and Technology (PennAITech) Collaboratory for Healthy Aging is to identify, develop, evaluate, commercialize, and disseminate innovative technology and artificial intelligence (AI) methods and software to support older adults and those with Alzheimer’s Disease (AD) and Alzheimer’s Disease and Related Dementias (ADRD) in their home environment. The Collaboratory is motivated by the need for a comprehensive pipeline from technology-based monitoring of older adults in the home, collection and processing monitoring data, integration of those data with clinical data from electronic health records, analysis with cutting-edge AI methods and software, and deployment of validated AI models at point of care for decision support. A central focus of the PennAITech Collaboratory is to advance this vision through the solicitation, review, and funding of pilot grants focused on technology and AI development to advance the science of care management and aging in place for vulnerable older adults or those with AD/ADRD receiving skilled home and community-based services. Funded pilot projects are supported through cores focused on administration, stakeholder engagement, technology identification and training, clinical translation and validation, networking, and ethical and policy issues. We will present our funded portfolio of funded projects by academic sites, industry and health systems, ranging from robotic tools for home assistance to predictive algorithms to support diagnostic excellence, smart home solutions and generative AI tools to facilitate coping and mental health services for older adults and their caregivers.

